# Shilling Attacks Detection in Recommender Systems Based on Target Item Analysis

**DOI:** 10.1371/journal.pone.0130968

**Published:** 2015-07-29

**Authors:** Wei Zhou, Junhao Wen, Yun Sing Koh, Qingyu Xiong, Min Gao, Gillian Dobbie, Shafiq Alam

**Affiliations:** 1 College of Computer Science, Chongqing University, Chongqing, China; 2 School of Software Engineering, Chongqing University, Chongqing, China; 3 Department of Computer Science, University of Auckland, Auckland, New Zealand; Hangzhou Normal University, CHINA

## Abstract

Recommender systems are highly vulnerable to shilling attacks, both by individuals and groups. Attackers who introduce biased ratings in order to affect recommendations, have been shown to negatively affect collaborative filtering (*CF*) algorithms. Previous research focuses only on the differences between genuine profiles and attack profiles, ignoring the group characteristics in attack profiles. In this paper, we study the use of statistical metrics to detect rating patterns of attackers and group characteristics in attack profiles. Another question is that most existing detecting methods are model specific. Two metrics, Rating Deviation from Mean Agreement (*RDMA*) and Degree of Similarity with Top Neighbors (*DegSim*), are used for analyzing rating patterns between malicious profiles and genuine profiles in attack models. Building upon this, we also propose and evaluate a detection structure called *RD-TIA* for detecting shilling attacks in recommender systems using a statistical approach. In order to detect more complicated attack models, we propose a novel metric called *DegSim’* based on *DegSim*. The experimental results show that our detection model based on target item analysis is an effective approach for detecting shilling attacks.

## 1 Introduction

Recommender systems have become an effective tool to recommend movies, music, news, books, research articles, social tags, and other items, and have played an important role in many popular websites, such as Amazon, YouTube, Netflix, and Yahoo!. Recommender systems predict a rating or preference that a user would give to an item. In general, recommender systems produce recommendations using two approaches [1, 2]. The first approach is collaborative filtering (*CF*). *CF* approaches typically build models from a user’s past behaviour coupled with similar decisions made by other users. This is then used to build a model to predict items or ratings for items that a user may be interested in. The second is content-based filtering, which uses the characteristics of an item to recommend additional items with similar properties. A key advantage of recommender systems using a *CF* approach is that it does not rely on the ability of the algorithms to analyse its content and thus is capable of recommending a variety of items, such as movies, without requiring a deep understanding of the content of the item itself [3, 4]. In contrast, by using a content-based filtering approach we may need additional information such as genre and actors. *CF* based recommender systems compare the collected data from a user to similar and dissimilar data collected from other users and calculates a list of recommended items for the user.

However, due to the open nature of *CF* recommender systems, they suffer vulnerabilities of being attacked by malicious users by injecting profiles consisting of biased ratings [5]. These attacks are carried out in order to influence the system’s behavior, and have been termed “shilling” or “profile injection” attacks, and attackers as shillers [4]. Some extraordinary measures have be introduced to increase the effort required to create profiles, for example, verification code that is required to be filled before a rating is made or an increase in the cost of creating a user account. These methods reduce the number of attack profiles, but also discourage participation thus decreasing the user engagement. There is the possibility of an attacker launching an attack as long as ratings can be made in recommender systems.

There may be a monetary incentive when an item is rated highly on a recommendation list. In some e-commerce websites, there are a team of shillers who can push a specified item to the recommended list in a short period of time for money [1]. Individuals may be interested in promoting or demoting an item, known as a target item, by manipulating the recommender system. Most attacks can be implemented as follows. The attacker takes on different identities within the system, and creates a user profile for each identity, which is referred as attack profiles. Within each of the profiles created, the attacker would then manipulate the recommendation by rating or recommending a particular target item. In order to obfuscate themselves and appear as genuine users in the system, the attack profiles will contain ratings for non-target items. These ratings can be selected in different ways either randomly or more intelligently if the attacker has prior knowledge of the ratings in the system. The attacker can manipulate the system into producing a desired recommendation behaviour. Recent work has shown that even modest attacks are sufficient to manipulate the behaviour of the most commonly used recommendation algorithms [6].

There are several hazards of attacks in recommender systems. Attacks can cause different losses to unprotected systems depending on the purpose of the attackers. The first is it will be unfair representation of users in recommender systems. The second is that the recommender systems failed to produce proper recommendations to users. Thus ruin the reputation of recommendation systems. Under some conditions, a large number of attack profiles can lead to a breakdown of a system [7]. It is difficult to prevent unscrupulous users from injecting fake data (profiles) into a system. To ensure the trustworthiness of recommender systems, attack profiles need to be detected and removed accurately.

The main contribution of this paper is a proposed hybrid attack detection structure, *RD-TIA*, which uses group features of attacks and target item analysis method. Two extended algorithms based on the *RD-TIA* detecting structure, *RD-TIA(a)* and *RD-TIA(b)* are proposed to detect different attack models. The second contribution of this research is that we proposed a novel metric *DegSim*′ based on *DegSim* to detect complex attack model attacks in recommender systems. The rest of the paper is organized as follows. In the next section, we examine previous work in the area of attack detection in recommender systems and background; in the Section 3 we describe the details of our approaches. Our experimental results are presented in Section 4. We discuss and summarize our research in Section 5.

## 2 Related work

The word “shilling” was first coined by [4]. There have been some recent research efforts aimed at detecting and reducing the effects of profile injection attacks [6, 8–13]. These attacks consist of a set of attack profiles, each containing biased rating data associated with a fictitious user identity. Since “shilling” profiles look similar to genuine profiles, it is difficult to identify them. Many attack profiles are based on random and average attack models which were introduced originally in [4] and used in [14]. Both of these attack models involve the generation of attack profiles using randomly assigned ratings to the filler items in the profiles. In a random attack the assigned ratings are based on the overall distribution of user ratings in the dataset, while in an average attack the rating for each filler item is computed based on its average rating for all users. In addition to these standard attack models, several more sophisticated models have been studied. Intentional attacks can cause the recommender system to become unreliable and untrustworthy, which can result in user distrust. In this section we concentrate on research in attack detection in a *CF* recommender system. There are three categories of attack detection algorithms: supervised, unsupervised, and semi-supervised.

In the first category, attack detection techniques are modelled as a classification problem. A lot of research has been undertaken to employ supervised learning for shilling attack detection [5, 15, 16]. Three classification algorithms, *k*NN-based, C4.5-based and *SVM*-based, are used to improve the robustness of the recommender system in [17]. These supervised algorithms need a large number of labeled users to enhance the accuracy. Classification-based methods require balanced numbers of attack and normal profiles to train the classifiers. Most early detection algorithms exploited signatures of attack profiles. These techniques were considered less accurate, since they looked at individual users and ignored the combined effect of such malicious users. Moreover, these algorithms do not perform well when the attack profiles are obscured. Some of these techniques use nearest neighbours classifiers, decision tree methods, rule based classifiers, Bayes classifiers, Neural Network classifiers, or *SVM* based classifiers [18–20].

In the second category, unsupervised detection approaches address these issues by training on an unlabeled dataset. These methods involve far less computational effort as compared to supervised approaches. The benefit of this is that these techniques facilitate online learning and improve detection accuracy. There has been significant research interest focused on detecting attack profiles using the unsupervised approach. Some of the techniques use clustering, association rules methods and statistical approaches [21–23]. Zhang et al. [23] used a Singular Value Decomposition (*SVD*) method to learn a low-dimensional linear model. Hurley et al. [21] utilizes Neyman-Pearson theory to construct both supervised and unsupervised detectors. An unsupervised shilling attack detection algorithm using principal component analysis (*PCA*) was proposed in [24]. Statistical detection techniques [7] are also used to detect profile injection attacks.

In the third category, semi-supervised detection approaches, such as [25, 26], make use of both unlabelled and labelled user profiles for multi-class modelling. Cao et al. [25] proposes a new semi-supervised method called *Semi-SAD* shilling attack detection algorithm using both types of data. HySAD introduces MC-Relief to select effective detection metrics, and semi-supervised Naive Bayes (SNB*λ*) to precisely separate random-filler model attackers and average-filler model attackers from normal users.

### 2.1 Attack Models

An attack consists of attack profiles that are introduced into the system in order to alter recommendation lists of a set of target items. Based on different assumptions about the attacker’s knowledge and purpose, a number of attack models have been identified, as described in [5].

There are four popular attack models in recommender systems: random attack, average attack, bandwagon attack, and segment attack models. Ratings in an attack profile can be divided into three sets of items: a target item *I*_*T*_; a selected item *I*_*S*_, selected set is a set of widely popular items or items that have common features, which is usually used to perform group attacks; and a set of filler items usually randomly chosen *I*_*F*_, filler items in a malicious profile are a set of items that make the profile look normal and makes a malicious profile harder to detect. Features of the attack models are shown in Table 1.

**Table 1:** Features of the attack models.

Attack model	*I*_*S*_(Selected Items)	*I*_*F*_(Filler Items)	*I*_*T*_(Target Items)
**Random Attack**	∅	random ratings	*r*_*max*_/*r*_*min*_
**Average Attack**	∅	mean of each item	*r*_*max*_/*r*_*min*_
**Bandwagon Attack**	*r*_*max*_	random ratings	*r*_*max*_/*r*_*min*_
**Segment Attack**	*r*_*max*_	random ratings	*r*_*max*_/*r*_*min*_

The quality of the filler items depends on the existing knowledge gathered from the recommender system. As more knowledge is obtained, an attack generated is more sophisticated. The major difference of attack models is how the ratings of filler items and the selected items are determined. The differences among attack models are the variance rating distribution in filler items and the selected items.

Random attack model is a naive attack in which the injected profile rates the set of randomly chosen fillers using a normal distribution and the standard deviation around the average rating of the system, as described in [14]. They then rate the set of target items with the maximum or minimum allowable rating based on the purpose of the attack. For example if the rating scores for a recommender system is between 1 and 5, where 1 represents an unfavourable rating and 5 represents a favourable rating, an attacker would rate the target item at 5 for a push attack and rate the target item at 1 for a nuke attack.

Average attack model is a more sophisticated attack model than random attack model and requires knowledge of the average rating of each item in the recommender system. Attackers rate items in the filler set randomly using a normal distribution with average set to the average rating of the filler items being rated and the standard deviation, as described in [14]. By introducing the average attack model, attackers disguise themselves and are harder to differentiate when compared to genuine users, thus, have a larger effect on recommendations. As with the random attack model, the ratings of target items are set to either the maximum or minimum allowable rating based on the purpose of the attack.

In addition to random and average attack models, several more sophisticated models have been studied [7]. In this work we have evaluated two other models, the bandwagon and segment attacks. Attackers choose items that many users have rated as selected items, in order to make attack profiles similar to genuine profiles. These profiles have a good probability of being similar to a large number of genuine profiles, since the high visibility items are those that many users have rated. Segment attack and bandwagon attack with different selected sets can be seen as group attacks. The principle behind the group attack, is that the best way to increase the $\frac{cost}{benefit}$ of an attack is to target one’s effort to those already predisposed towards one’s product. In other words, it is likely that an attacker wishing to promote a particular item will be interested not in how often it is recommended to all users, but how often it is recommended to likely users. The segment attack model is designed to push an item to a targeted group of users with known or easily predicted preferences. In the bandwagon attack model, the attacker using Zipf’s law will build attack profiles containing those items that have high visibility. Such profiles will have a good probability of being similar to a large number of users, since the high visibility items are those that many users have rated.

### 2.2 Detecting Metrics

Attack profiles differ from that of genuine profiles in a statistical way. There are two main differences. The former is the rating given to the target item (items); the latter is the rating distribution among the filler items. There are different metrics that have been proposed by [14, 27] to measure the similarity of differences. In this section we will look at two metrics, *RDMA* and *DegSim*.

*RDMA* measures the deviation of agreement from other users on a set of target items, combined with the inverse rating frequency for these items. *RDMA* can be calculated in the following way:

$$\begin{array}{c} RDMA_{u}=\frac{\sum_{i=0}^{N_{u}}\frac{\left|r_{u,i}-\bar{r_{i}}\right|}{NR_{i}}}{N_{u}} \end{array}$$ (1)

where *N*_*u*_ is the number of items user *u* rated, *r*_*u*,*i*_ is the rating given by user *u* to item *i*, *NR*_*i*_ is the overall number of ratings in the system given to item *i*.

**Fig 1:**
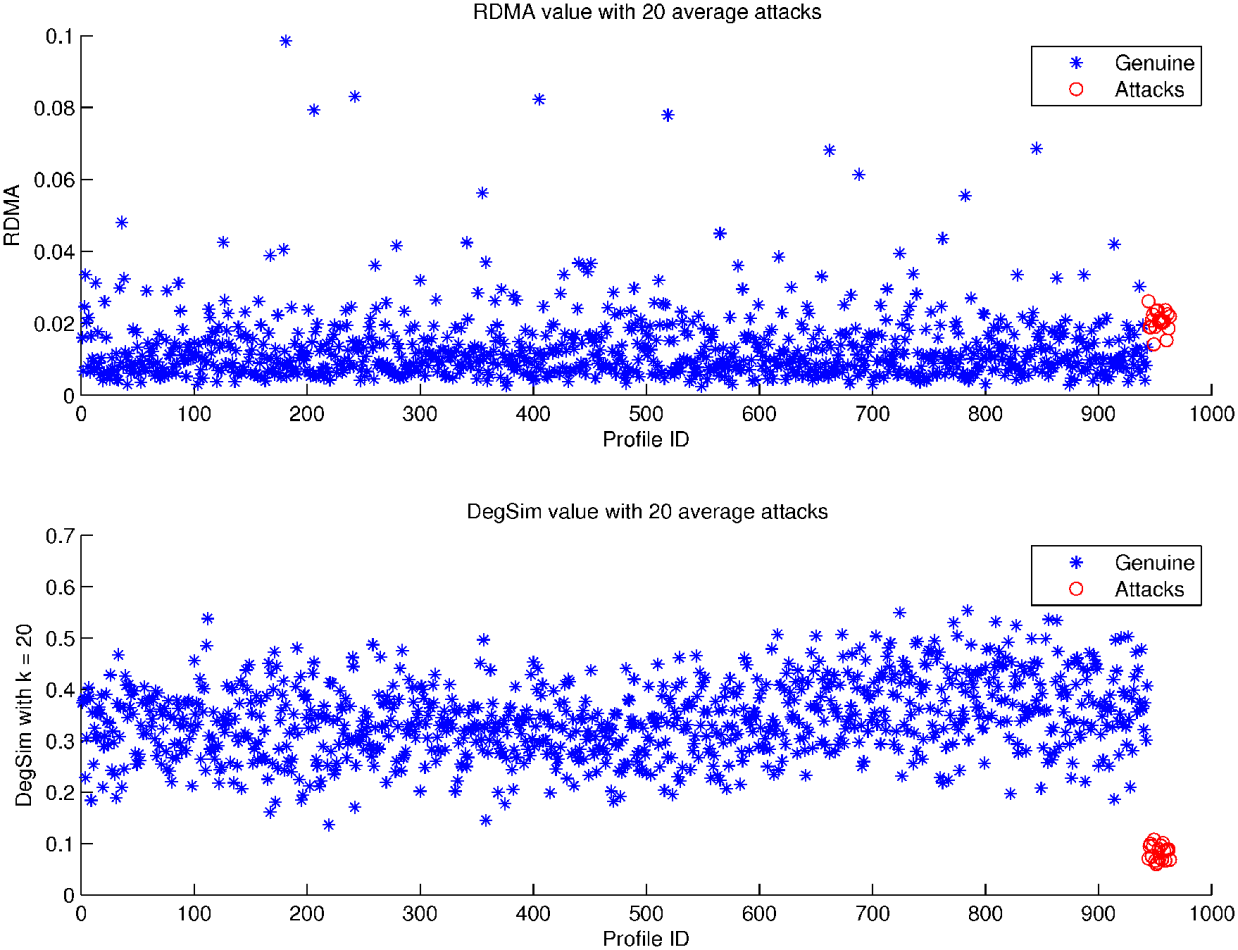
RDMA and DegSim value distribution with average attacks. RDMA Metric value and DegSim Metric value of each profile of MovieLens 100k Dataset.

The *DegSim* attribute is based on the average Pearson correlation of the profile’s *k* nearest neighbours and is calculated as follows:

$$\begin{array}{c} DegSim=\frac{\sum_{u=1}^{k}W_{uv}}{k} \end{array}$$ (2)

where *W*_*uv*_ is the Pearson correlation between user *u* and user *v*. In general the value of *k* can be easily determined for most datasets, as we can measure the degree of separability using different separable extension techniques used in computational geometry. Fig 1 shows the *RDMA* and *DegSim* value distribution in the random attack model, with an attack size of 20, filler size is 5%, and *k* = 20 in *DegSim*.

## 3 Detecting Profile Injection Attacks

In this section, a hybrid two-phase detection structure *RD-TIA* is proposed. *RD-TIA* is based on profile feature extraction and target item analysis. Two shilling attack methods, *RD-TIA(a)* and *RD-TIA(b)* are proposed based on the detection structure. *RD-TIA(a)* is used to detect random attacks and average attacks. Considering profile features of group attack models are different, a new metric is proposed to extract profile features of group attack models. *RD-TIA(b)* is proposed to detect group attacks.

### 3.1 A Hybrid Detection Structure (*RD-TIA*)

In order to get a better the $\frac{cost}{benefit}$ in an attack, overall attackers would have a high influence in the system in order to promote the target items effectively. However, there are three different features in attack profiles, which enable us to differentiate between genuine and attack profiles. In this section, we propose a structure to detect shilling attacks using statistical metrics.

**Fig 2:**
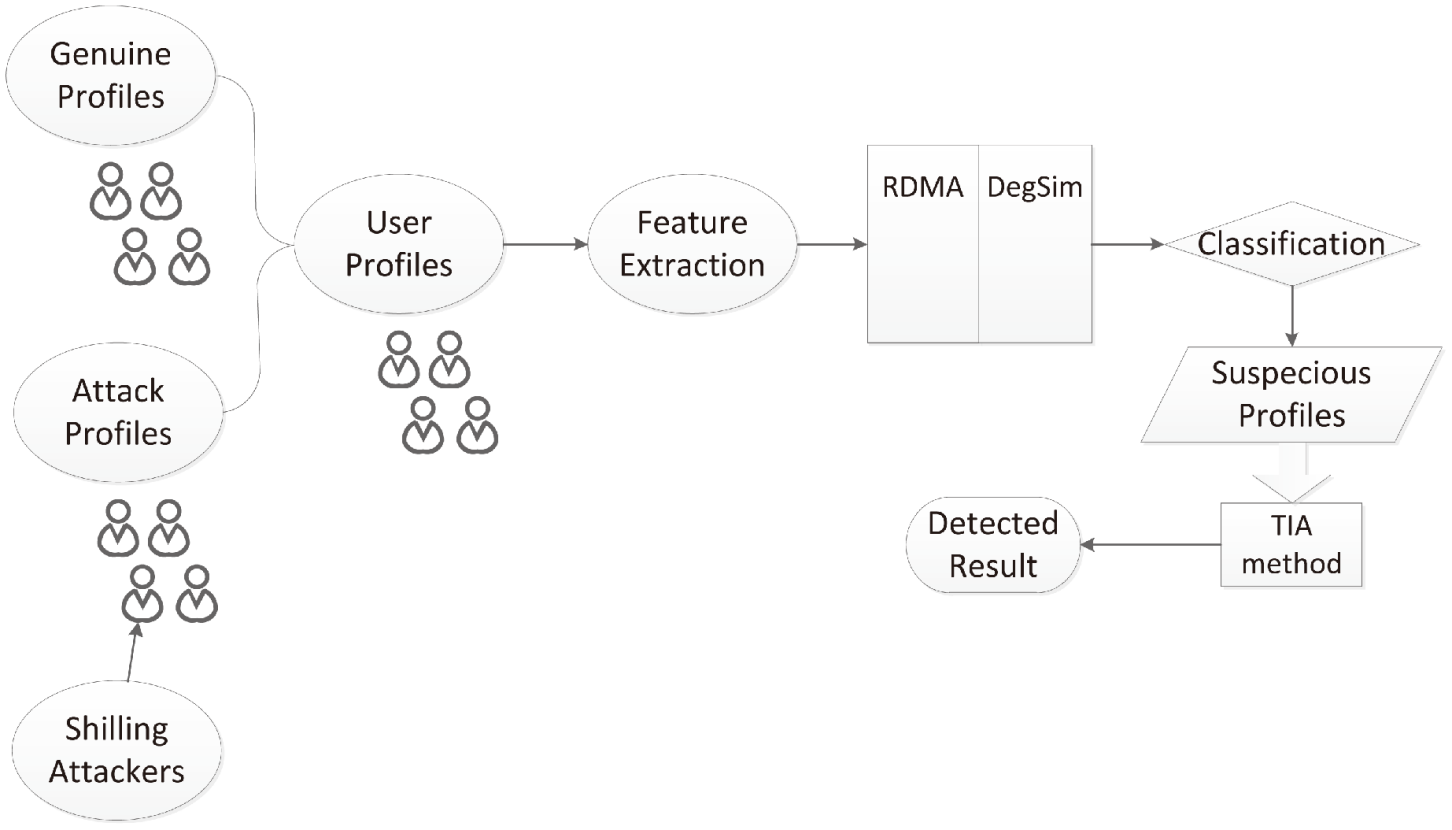
RD-TIA Detecting structure based on two metrics RDMA and DegSim. There are two phases in RD-TIA. In the first phase, extract profile attributes and determine the suspicious profiles by using two statistical metrics DegSim and RDMA.

Firstly in attack profiles, filler items are randomly chosen thus the similarity based on these filler items between attack and genuine profiles should be lower. We choose 20 neighbours that have the highest similarity in *DegSim*. Secondly, since shilling attacks usually try to push items with low ratings or vice versa in nuke attacks, the users mounting such an attack will assign a rating that deviates from the average rating value assigned by the genuine profiles. We use metric *RDMA* to calculate this. Attackers’ profiles should therefore have relatively high values for *RDMA*, as well as very low values in *DegSim*. Fig 1 shows the distribution of *RDMA* and *DegSim* when average attacks are injected. Last but not least, all target items are assigned a highest or lowest value, the count number of this value should be bigger than other values among items. Based on these three reasons, we propose a detection structure called *RD-TIA* that uses two metrics, *RDMA* and *DegSim* to reveal these distinctive features in the rating patterns. Feature extraction using Eqs (1) and (2). Profiles that have a greater value in *RDMA* and smaller *DegSim* value are suspected of being attack profiles. Since there must be some false positives in the detecting result. Based on the third reason, we proposed a Target Item Analysis (*TIA*) method to filter genuine profiles out. The detection model is shown in Fig 2.

**Fig 3:**
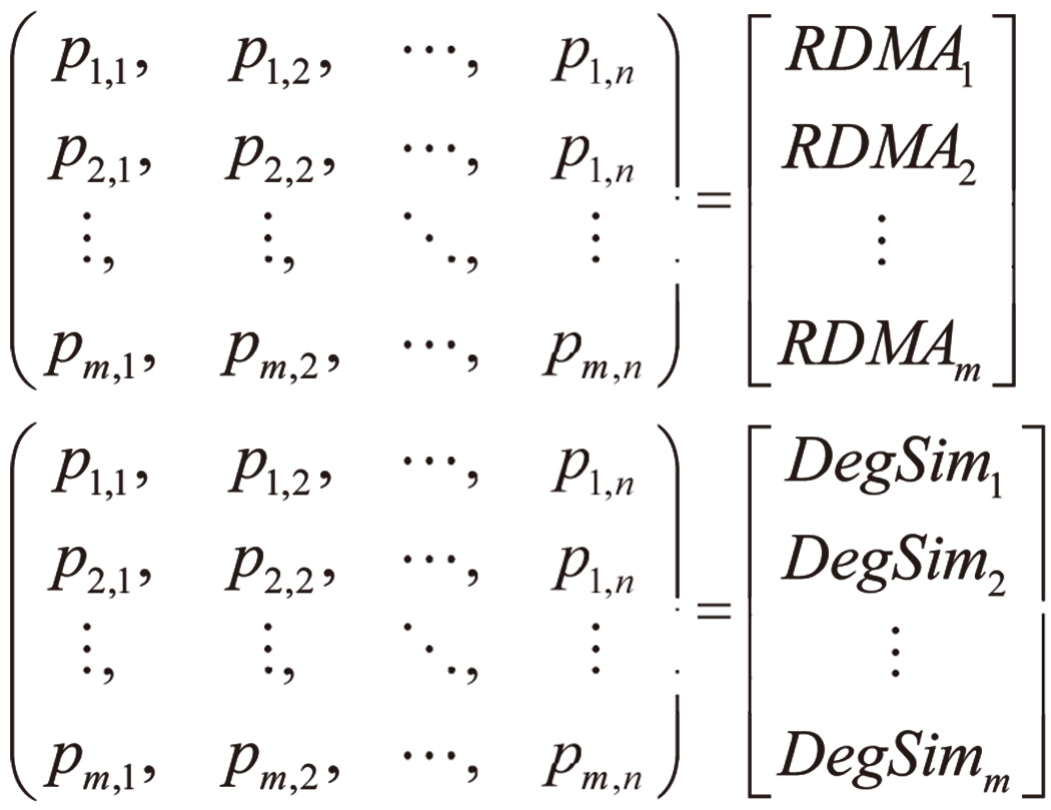
RD-TIA Detecting structure based on two metrics RDMA and DegSim. There are two phases in RD-TIA. In the first phase, extract profile attributes and determine the suspicious profiles by using two statistical metrics DegSim and RDMA.

There are two phases in *RD-TIA*. In the first phase, we extract profile attributes using Eqs (1) and (2), as shown in Fig 3; determine the suspicious profiles by using two statistical metrics, *DegSim* and *RDMA*. From this process, we get a pool of suspicious profiles *SUS*_*RD*_. The pseudocode is shown in Algorithm 1.

**Algorithm 1** *RD-TIA* Phase 1: Find suspicious profiles

**Input**: Mixed genuine and shilling rating Matrix M;

**Output**: Suspected profiles, *SUS*_*RD*_;

 1: *RDMA*_*u*_ ← Calculate *RDMA*(*M*);

 2: *DegSim*_*u*_ ← Calculate *DegSim*(*M*);

 3: {*R*_1_, *R*_2_} ← Classify(*RDMA*_*u*_);

 4: {*D*_1_, *D*_2_} ← Classify(*DegSim*_*u*_);

 5: *D* = max(*D*_1_, *D*_2_), *R* = max(*R*_1_, *R*_2_);

 6: *SUS*_*RD*_ ← {*SUS*_*RD*_ ∣*D* ∩ *R*};

 7: **return** suspected profiles *SUS*_*RD*_.

In the second phase, we use the *TIA* method to filter out genuine profiles. Items that could be suspicious target items in the *SUS*_*RD*_ *Set* from Phase 1 are found. An item is considered a target item, when it is rated with maximum or minimum score *r* proportionally higher than the rest. For example, if 80% of the profiles in *SUS*_*RD*_ rated an item with the highest (or lowest) score, we consider that item as a target item. The intuition behind this is that we believe that the attackers will have specific target items that they target when they commit an attack. They would rate target items with the highest or lowest possible rating depending on the type of attack. To detect the proportion, we use an absolute count threshold *θ*. If count(*r*) of *Item*_*i*_ is greater than *θ*, then *Item*_*i*_ is regarded as a target item, and the profiles that rated *Item*_*i*_ with the highest rating are considered as attackers. We choose the threshold value *θ* based on the assumption that if attackers want to make a considerable prediction shift, which is described in [4, 28], to the system and push a target item up, a certain number of injected attack profiles are required. An average size attack requires the number of attack profiles injected to be greater than 20 for a prediction shift and *MAE* shift, as shown in [4]. From this assumption we can calculate the upper bound threshold that is necessary for a shift to occur. To be conservative we chose a *θ* of 6 in case of small scale attacks. The pseudocode in Algorithm 2 shows how we filter out genuine profiles.

**Algorithm 2** *RD-TIA* Phase 2, Filter out genuine profiles.

**Input**: The set of suspected profiles *SUS*_*RD*_; highest rating *r*; item set *I*;

**Output**: Final detect result set *DetectedResult*;

 1: *DetectedResult* = ∅;

 2: ∀*i* ∈ *I*, *count*_*i*_ ← number of ratings in *item*_*i*_ equal to *r*;

 3: **While** max(*count*) > *θ* **do**

 4: *item*_*t*_ ← {*item*_*i*_∣*count*_*i*_ = max(*count*)};

 5: ∀*p* ∈ *SUS*_*RD*_, *P* ← *p* rate *item*_*t*_ with *r*;

 6: *DetectedResult* ← *P*∪*DetectedResult*;

 7: *SUS*_*RD*_ ← *SUS*_*RD*_ − *P*;

 8: **end while**

 9: **return** *DetectedResult*.

Let us take the push attack as an example. Consider Table 2 as the *SUS*_*RD*_ *Set* we obtained from in Phase 1. Each row in the matrix is the rating for the *m* items by a user. Table 2 shows genuine user profiles from *User*_1_ to *User*_*m*_ and attackers profiles from *Attacker*_1_ to *Attacker*_*p*_. The last row is the count number of rating 5, in this example, *Item*_5_ is the target item. In the example, *Attacker*_1_ to *Attacker*_*p*_ and *User*_3_ are considered as attacks.

**Table 2:** An example of rating matrix and attack profiles.

	Item_1_	Item_2_	Item_3_	Item_4_	Item_5_	….	Item_*n*_
User_1_	5	2	3	0	0	….	5
User_2_	2	0	4	1	2	….	3
User_3_	4	2	3	0	5	….	0
User_4_	0	3	0	3	4	….	3
….	….	….	….	….	….	….	….
User_*m*_	2	0	4	1	2	….	3
Attacker_1_	2	1	0	0	**5**	….	4
Attacker_2_	2	2	0	0	**5**	….	3
Attacker_3_	1	2	0	0	**5**	….	2
….	….	….	….	….	….	….	….
Attacker_*p*_	2	0	0	0	5	….	4
Count(5)	2	2	2	2	9	….	3

Considering different attack models, we proposed two methods based on the proposed attack detection model *RD-TIA*. The first one *RD-TIA(a)* is used to detect random attacks and average attacks. The second one *RD-TIA(b)* is used to detect segment attacks and bandwagon attacks. The main difference between the two methods is that they use different classification methods to split profiles into genuine profiles and suspected profiles in Phase 1.

### 3.2 Detecting Random and Average Attacks (*RD-TIA(a))*

In this section, we proposed a detection method *RD-TIA(a)* based on the *RD-TIA* structure to detect random and average attacks. As discussed earlier, there is a feature in random and average attack that differentiate these two attacks. The *RDMA* value of attack profiles is relatively higher than that of genuine profiles, and the *DegSim* value of attack profiles is relatively smaller than that of genuine profiles. So we can consider profiles that have higher *RDMA* values and lower *DegSim* values as attack profiles. In the first phase in *RD-TIA(a)*, we determined the suspicious profiles by using two statistical metrics *RDMA* and *DegSim*. In this phase, the *RDMA* value for each profile is calculated. If the *RDMA* value for a profile *u* is above a maximum *ϵ*_*RDMA*_ threshold then we consider this profile as a suspicious profile (*SP*_*RDMA*_).

$$SP_{RDMA}=\{u|RDMA_{u}\geq \epsilon_{RDMA}\}$$

From this process, we get a pool of suspicious profiles, *SP*_*RDMA*_, that had *RDMA* values above the assigned threshold. We also calculate the *DegSim* value for each of the profiles in the same way. If the *DegSim* value for a profile *u* is below a minimum *ϵ*_*DegSim*_ threshold then we consider this profile as a suspicious profile (*SP*_*DegSim*_).

$$SP_{DegSim}=\{u|DegSimA_{u}\leq \epsilon_{DegSim}\}$$

From this process, we get a pool of suspicious profiles, *SP*_*DegSim*_, that had *DegSim* values below the assigned threshold. Lastly we consider the intersection between the pool of *SP*_*RDMA*_ and *SP*_*DegSim*_, as our *SUS*_*RD*_.

$$SUS_{RD}=SP_{DegSim}\cap SP_{RDMA}$$

We set generous thresholds *ϵ*_*RDMA*_ and *ϵ*_*DegSim*_, allowing more profiles to be considered as suspicious and then filter out the misclassified profiles in the second phase.

### 3.3 Detecting Group attacks (*RD-TIA(b))*

In this section, we use the *RD-TIA* detection model to determine more complex attack models. There are two major differences between *RD-TIA(b)* and *RD-TIA(a)*. The first is the metric used, the other is classification method used. As described earlier, the selected set in attack profiles can make attack profiles more complicated than random and average attacks. It is hard to classify profiles if attackers adjust the selected set in the first phase of the algorithm. Thus we proposed a new metric call *DegSim*′ that calculates the similarity of rating independently. The following assumption is used: the *DegSim* value of genuine profiles follow a Gaussian distribution, and the value of each rating score also follows the Gaussian distribution. If one rating score does not follow a Gaussian distribution, we adjust value of these profiles such that it is larger than genuine ones. *DegSim*′ can be calculated using Eq (3):

$$\begin{array}{c} DegSim^{\prime }=\sum_{r\in R}|DegSim_{r}-\bar{DegSim_{r}}| \end{array}$$ (3)

where *R* is the rating scale of the rating database and *r* is a rating score, and $\bar{DegSim_{r}}$ is the mean value of *DegSim*_*r*_. When calculating *DegSim*_*r*_, all ratings not equal to *r* are replaced with 0, where 0 means unrated. *DegSim*_*r*_ is then calculated with Eq (2) using the new transformed rating matrix. This method reshape *DegSim*′ rating score of attack profiles such that it is relatively higher than genuine profiles, as long as there are differences in each rating scale. Thus whilst there may be no difference between attack profiles and genuine profiles using *DegSim*, there would be differences if it was used in *DegSim*′. Using this method the *DegSim*′ rating score of attack profiles will be relatively higher than genuine profiles, when there are differences in the rating scale. Based on above-mentioned reasons, *RDMA* and *DegSim*′ are used to reveal these distinctive features in the rating patterns in *RD-TIA(b)*.

In the first phase of *RD-TIA(b)*, we multiply *RDMA*_*u*_ and $DegSim_{u}^{\prime }$ together, making low values lower, and high values greater in the product of *RDMA*_*u*_ and $DegSim_{u}^{\prime }$. We use *k-means* to split the product into two parts. Profiles in the higher part are probably attack profiles. From this process, we obtain a pool of suspicious profiles. Like in *RD-TIA(a)*, we then use the second phase to filer out genuine profiles.

## 4 Experiments and Discussions

In this section, we conduct extensive experiments on different datasets and benchmark detection methods. Experimental setup and metrics are introduced. Experimental results of the two detection methods *RD-TIA(a)* and *RD-TIA(b)* are introduced respectively, followed by a discussion.

### 4.1 Experimental Setup

We now describe datasets we have used in the experiments, and the metrics we have used to evaluate attributes, followed by experimental results. MovieLens datasets (http://grouplens.org/datasets/movielens/) published by GroupLens, are mainly used in the experiments. In order to show the scalability of the method, some other datasets are used, including a subset of Netflix dataset (http://www.netflixprize.com/) and a subset of Eachmovie dataset (http://grouplens.org/datasets/eachmovie/). Details of the four datasets is given in Table 3. For the two MovieLens datasets and the subset of the Netflix dataset, all ratings are integer values between 1 and 5, where 1 is the lowest (disliked) and 5 is the highest (liked). While in the Eachmovie dataset, all ratings are integer values between 1 and 6, where 1 is the lowest (disliked) and 6 is the highest (liked). We filter out users who have rated less than 20 movies.

**Table 3:** Datasets used in the experiments.

Dataset	ML100K	ML1M	Netflix	Eachmovie
#Users	943	6,040	4,334	2,000
#Movies	1,682	3,952	3,558	1,623
#Ratings	80,000	1,000,209	552,054	137,425
Sparseness	94.96%	95.81%	94.42%	95.77%
Rating scale	1 2 3 4 5	1 2 3 4 5	1 2 3 4 5	1 2 3 4 5 6

To evaluate the performance of our technique we used several metrics: *Detection Rate*, *False Positive rate*, *AUC*, *Sensitivity* and *Specificity*. Detection rate is defined as the number of detected attacks divided by the number of attacks.

$$\begin{array}{c} Detection\;Rate=\frac{\#Detection}{\#Attacks} \end{array}$$ (4)

False positive rate is the number of genuine profiles that are predicted as attacks divided by the number of genuine profiles.

$$\begin{array}{c} False\;Positive\;Rate=\frac{\#False\;Positives}{\#Genuine\;Profiles} \end{array}$$ (5)

*Sensitivity* and *Specificity* are defined in [29, 30].

$$\begin{array}{c} Sensitivity=\frac{\#True\;positives}{\#True\;positives+\#False\;Negatives} \end{array}$$ (6)

The *Specificity* measures the percentage of correctly identified genuine profiles (True Negatives), and the *Sensitivity* measures the percentage of correctly detected attack profiles (True Positives).

$$\begin{array}{c} Specificity=\frac{\#True\;Negatives}{\#True\;Negatives+\#False\;Positives} \end{array}$$ (7)

*AUC* is the area under the *ROC* curve. *ROC* is a graphical plot which is created by plotting false positive rate (*FPR*) and true positive rate *TPR*. *FPR* is the fraction of true positives out of the total actual positives and *TPR* is the fraction of false positives out of the total actual negatives. Accuracy is measured by the area under *ROC* curve. An area of 1 represents perfect results and an area of 0.5 represents insignificant results.

### 4.2 Performance of *RD-TIA(a)*

In this section, performance of *RD-TIA(a)* is introduced. Single-targeted item and Multi-targeted items attacks are detected using *RD-TIA(a)*. Single-targeted item detection, that is, there is only one target item in one attack. Multi-targeted items detection, that is, there are more than one target items in one attack. Performance of *RD-TIA(a)* is compared with a SVD-based attack detection.

In order to simulate real attacks in recommender systems, attack profiles generated by random and average attack models are injected. In the experiments, two different variables: the attack size and the filler size are varied. We vary the attack size from 2% to 14%. We also vary the filler size from 3% to 9%. The experiments in Section 4.2 are based on the ML100K dataset. In order to get the accurate result, we repeat our tests 100 times.

In choosing the threshold values of *RDMA* and *DegSim*, we would like to get values that have high separability, because it is easier to distinguish between genuine and attack profiles when there is high separability. We adjusted parameters so that it produced low false negatives and low false positives. In these experiments we noticed that the intervals between *DegSim* values for the profiles were smaller when compared to the intervals between *RDMA* values for the profiles.

We set the thresholds for *ϵ*_*RDMA*_ and *ϵ*_*DegSim*_ as:

$$\begin{array}{c} \epsilon_{DegSim}=\lambda \sum_{u=1}^{n}\frac{DegSim_{u}}{n} \end{array}$$ (8)

and

$$\begin{array}{c} \epsilon_{RDMA}=\gamma \sum_{u=1}^{n}\frac{RDMA_{u}}{n} \end{array}$$ (9)

We choose a different weight for *λ* and *γ*. In the experiments we carried out we used *λ* = 1 and *γ* = 0.6. Setting these weights we notice that the false negative rate is lower and there are fewer positives. As we pointed out previously the threshold value of *RDMA* and *DegSim* are generous thus allowing false negative profiles into the *SUS*_*RD*_ set.

#### 4.2.1 Single-Targeted Item Detection

There are four types of attack in single-targeted attacks, single random push, single average push, single random nuke and single average nuke. The false positive rate and *AUC* value of the four attack models are similar when the filler size varies, so we show the comparisons between attack models when the filler size is 5%.

**Fig 4:**
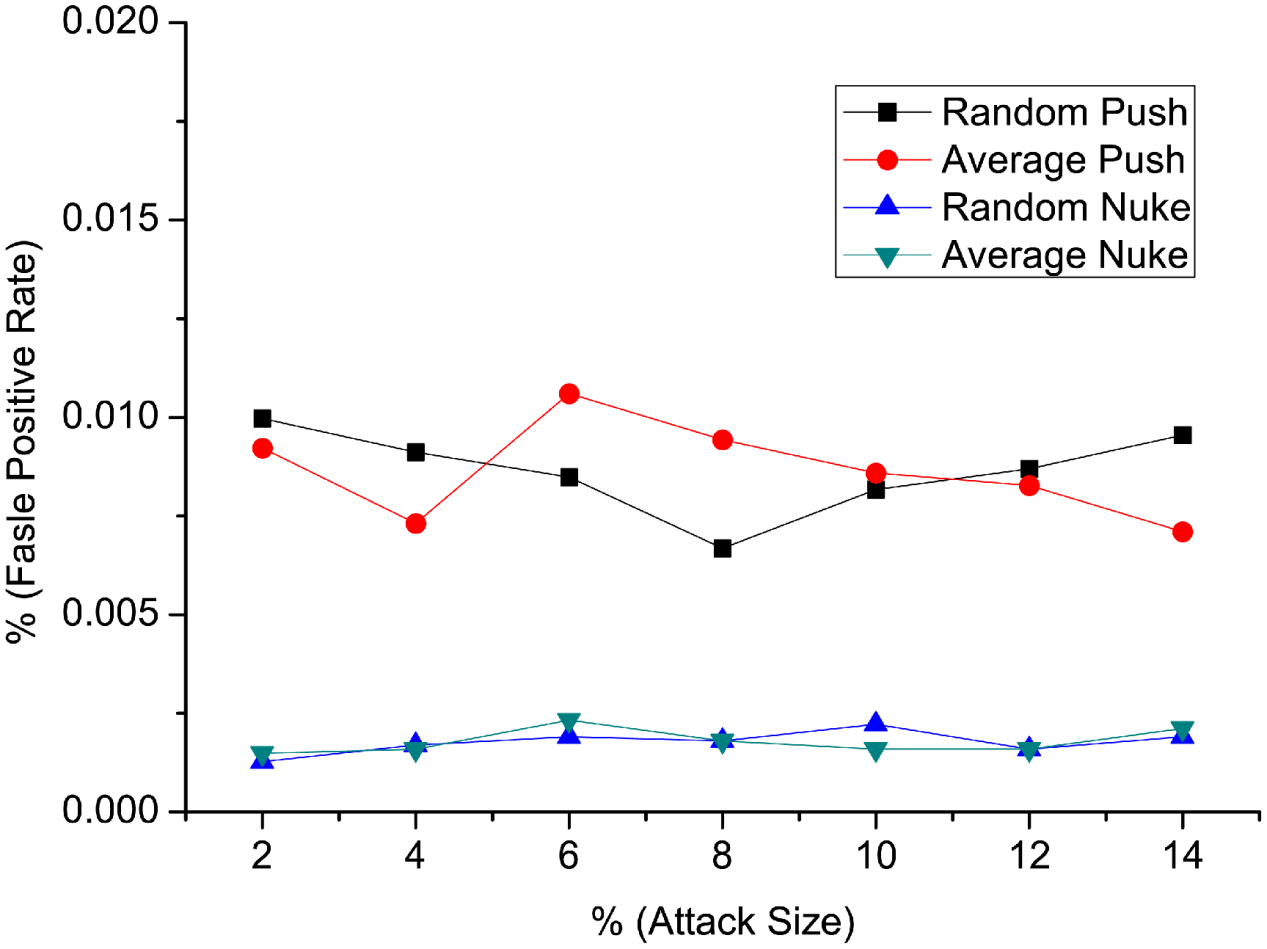
Comparison of false positive rate in single-targeted detection when attack size varies. There is only one target item in each attack, comparison of false positive rate in random push attack, average push attack, random nuke attack and average nuke attack.

**Fig 5:**
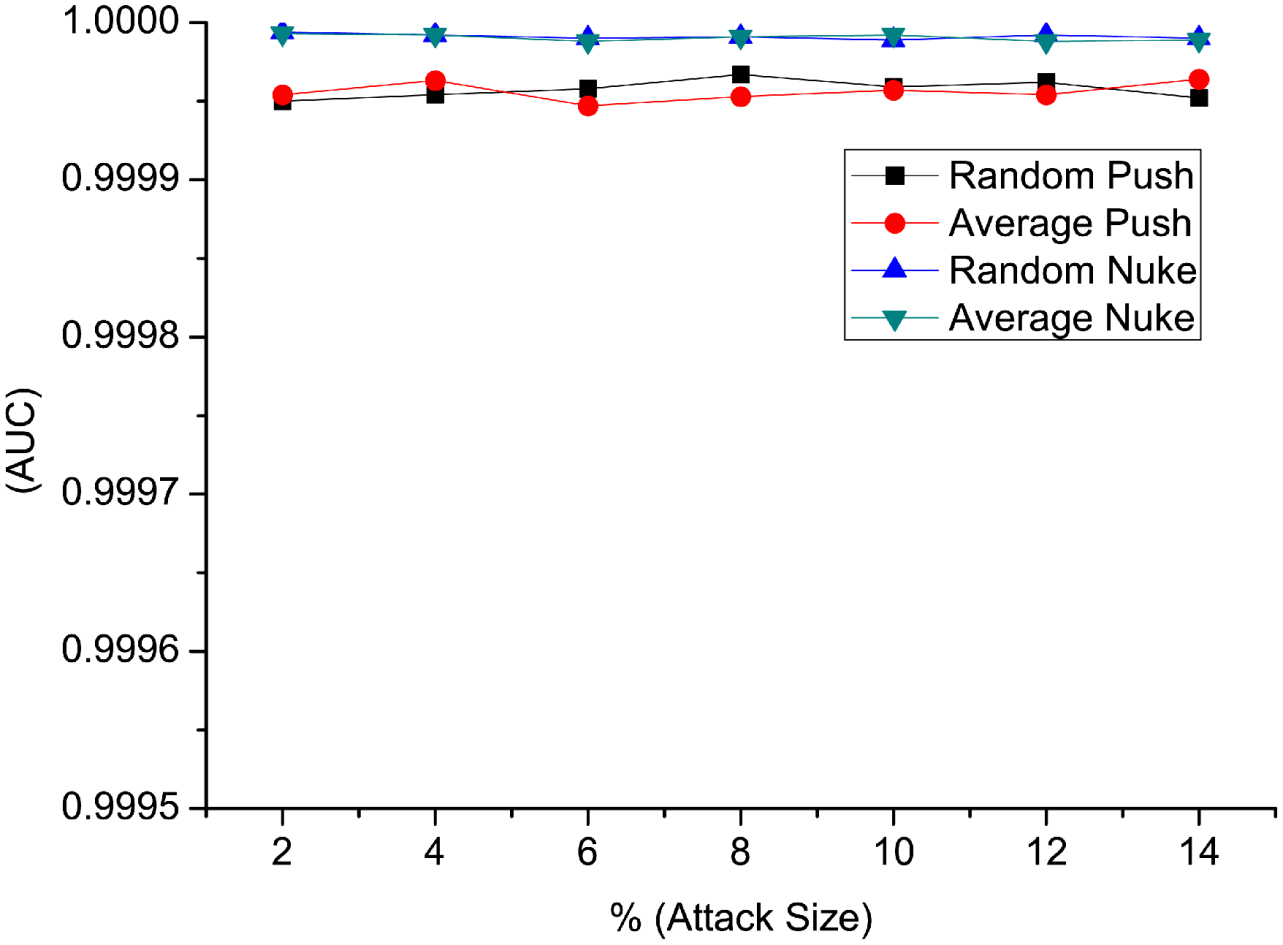
Comparison of AUC value in single-targeted detection when attack size varies. There is only one target item in each attack, comparison of AUC value in random push attack, average push attack, random nuke attack and average nuke attack.

Fig 4 and Fig 5 show the detection result of false positive rate and *AUC* when injecting random attacks and average attacks. We notice that the false positive rate in all four types of detection is smaller than 0.02%. The false positive rate remains stable as attack size changes, and there are no big changes when the filler size varies. On the other side, false positive rate in nuke attack types are lower than that of push attack types. Fig 5 shows the *AUC* values for different attack models. *AUC* values are greater than 0.9999, which is near to a perfect result. *AUC* values of nuke detection type is slightly higher than that of push detection type. In conclusion, the performance of *RD-TIA(a)* on random and average attacks is good with only one target item in one attack, with high detection rate and low false positive rate. Detection result of nuke attack is better than that of push attack using *RD-TIA(a)* in the same condition.

#### 4.2.2 Multi-Targeted Items Detection

In order to check the performance when the number of target items varies in one attack. A test is designed with the number of target items varies from 1 to 10, and 20 profiles for each target item. We also compare four attack models when filler size is 5%. We considered the result as false positive and *AUC* value when the numbers of targeted items are different to that of the ground truth.

**Fig 6:**
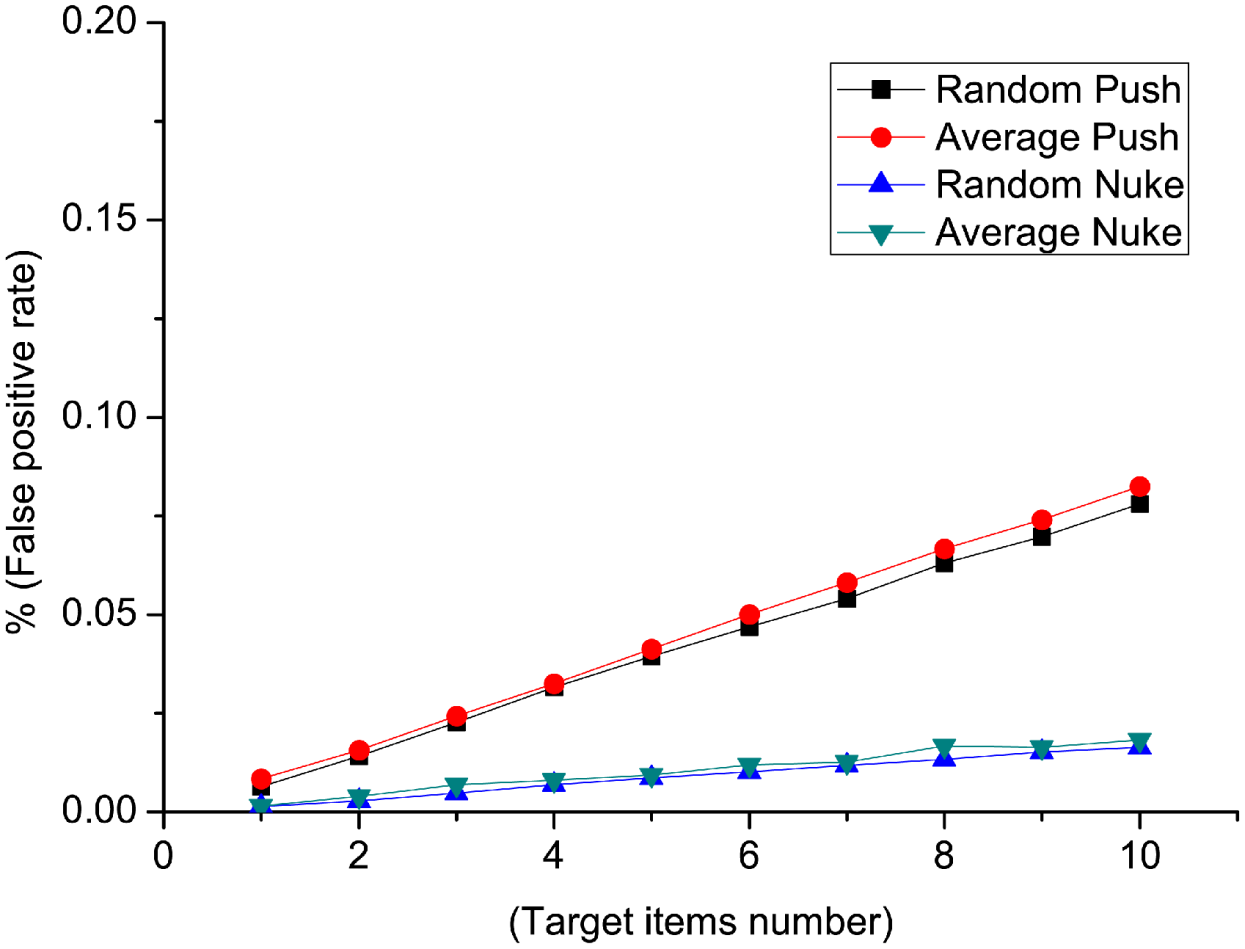
Comparison of false positive rate in multi-targeted detection when the number of target items varies. There are multi-target items in each attack. Comparison of false positive rate in random push attack, average push attack, random nuke attack and average nuke attack.

**Fig 7:**
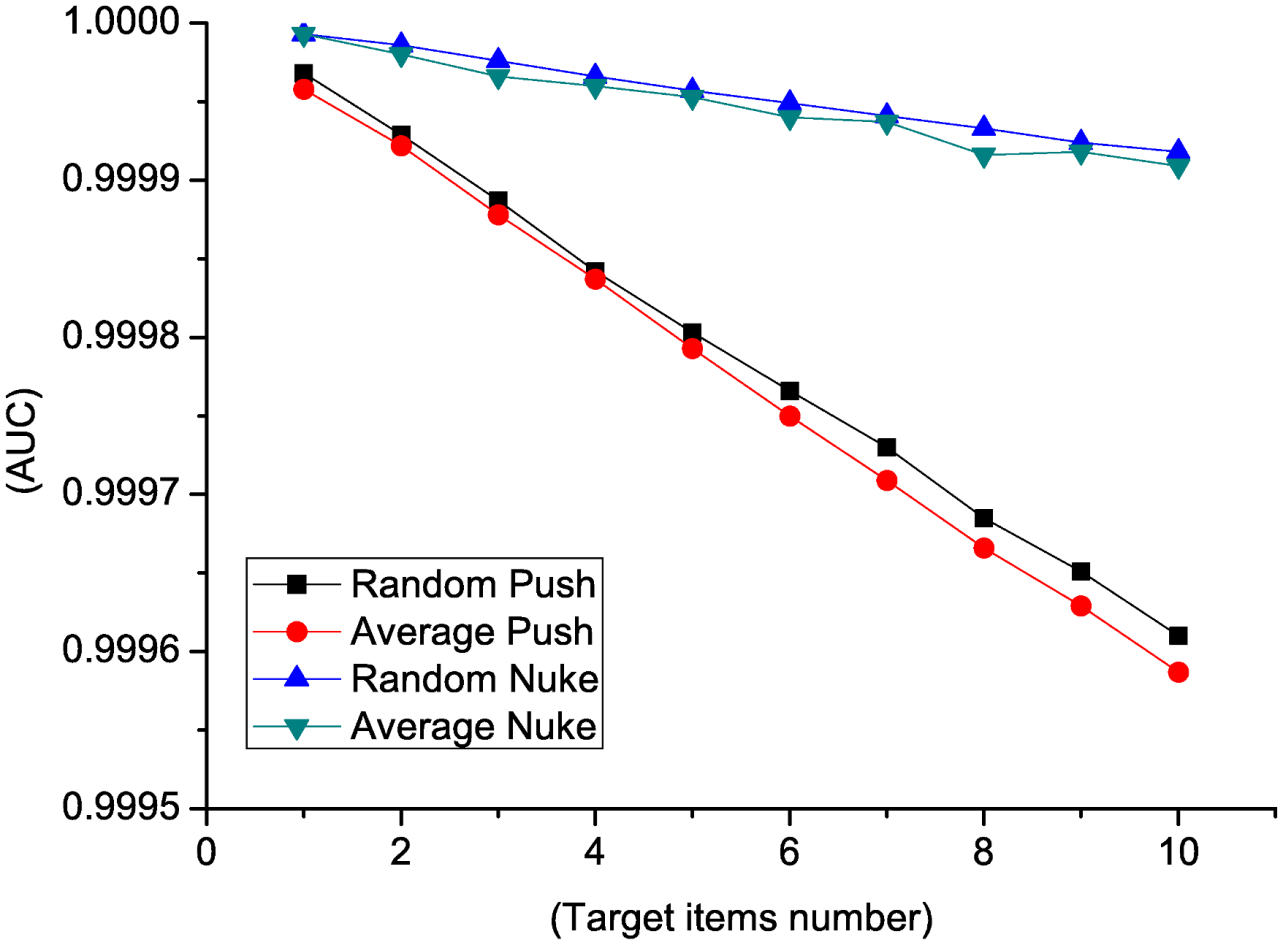
Comparison of AUC value in multi-targeted detection when the number of target items varies. There are multi-target items in each attack. Comparison of AUC value in random push attack, average push attack, random nuke attack and average nuke attack.

We notice in Fig 6 that the false positive rate increases when the number of target items increases. There is no big difference in false positive rate between random and average detection when the attacks are the same purpose. On the other side, the false positive rates of nuke attacks are smaller than that of push attacks. *AUC* values are compared in Fig 7 of different attack models. *AUC* values of different attack models decline when the number of target items increases. There is no big difference in *AUC* values between random and average detection when the attacks are the same purpose (push or nuke). In conclusion, the performance of *RD-TIA(a)* on random and average attacks with multi target items in one attack is not as good as single target items attack. Detection result of nuke attack is better than that of push attack using *RD-TIA(a)* in the same condition.

#### 4.2.3 Comparisons with SVD-based method

We compare our results of single random detection using *RD-TIA(a)* with a *SVD-based* algorithm in [28]. For a fair comparison we carry out the following experiments with the same parameter settings as the experiments used in the *SVD-based* method.

Table 4 shows the detection result of random attacks when the attack size varies. Three parameters are compared in the test, *AUC*, detection rate and false positive rate. *AUC* value of *SVD-based* method is better than that of *RD-TIA(a)*; False positive rate of *SVD-based* method decrease with attack size increases, while false positive rate of *RD-TIA(a)* is lower in push attack detection. False positive rate of *SVD-based* method in nuke attack detection is better than that of *RD-TIA(a)* when attack size is over 50. Detection rate of *RD-TIA(a)* is 100%, which is better than that of *SVD-based* method.

**Table 4:** Detection result of random attacks when the filler size is 3% and attack size varies.

		SVD-based method			RD-TIA		
Intent	Attack Size	AUC	Detection Rate	False Positive Rate	AUC	Detection Rate	False Positive Rate
Push	20	0.9999 ± 0.0001	99.25% ± 1.83%	0.22% ± 0.01%	**0.99999±0.00001**	**100%**	**0.003%±0.002%**
	50	0.9998 ± 0.0002	97.80% ± 1.82%	0.14% ± 0.02%	**0.99999±0.00001**	**100%**	**0.001%±0.001%**
	100	0.9999 ± 0.0001	96.95% ± 1.73%	0.07% ± 0.01%	**0.99999±0.00001**	**100%**	**0.002%±0.002%**
	200	0.9999 ± 0.0000	94.20% ± 1.41%	0.02% ± 0.01%	**0.99999±0.00001**	**100%**	**0.001%±0.001%**
Nuke	20	0.9999 ± 0.0001	94.20% ± 1.41%	0.22% ± 0.01%	**0.99996±0.00003**	**100%**	**0.01%±0.01%**
	50	0.9998 ± 0.0002	98.00% ± 1.72%	0.15% ± 0.02%	**0.99990±0.00006**	**100%**	**0.02%±0.01%**
	100	0.9999 ± 0.0000	97.75% ± 1.12%	0.06% ± 0.01%	**0.99966±0.00004**	**100%**	**0.07%±0.01%**
	200	0.9999 ± 0.0000	93.55% ± 1.86%	0.02% ± 0.01%	**0.99959±0.00001**	**100%**	**0.08%±0.00%**

Table 5 shows detection results of random push attacks with an attack size of 100 while varying rated filler items. *AUC* value of *RD-TIA(a)* method is better than that of *SVD-based*; False positive rate of *SVD-based* method decrease with filler size increases, while false positive rate of *RD-TIA(a)* increases when number of rated items increases. False positive rate of *RD-TIA(a)* is not as good when all items are rated. Detection rate of *RD-TIA(a)* is 100%, which is better than that of *SVD-based* method. Table 6 shows the detection result of random push attacks with an attack size of 100 and the number of target items varies. *AUC* value and detection rate of *SVD-based* method decrease when the number of target items increases. *RD-TIA(a)* gets better results than that of *SVD-based* method. Detection rate of *RD-TIA(a)* method is better than that of *SVD-based* method. *AUC* value of *RD-TIA(a)* method is higher than that of *SVD-based* method. False positive rate of *RD-TIA(a)* method is lower than that of *SVD-based* method.

**Table 5:** Detection result of random push attacks(with 100 bots) when the filler size varies.

	SVD-based method			RD-TIA		
Rated items	AUC	Detection rate	False Positive Rate	AUC	Detection Rate	False Positive Rate
20	0.9744 ± 0.0150	7.45% ± 4.72%	0.17% ± 0.02%	**0.99994±0.00017**	**99.99%**	**0.003%±0.002%**
50	0.9941 ± 0.0030	46.35% ± 8.97%	0.10% ± 0.01%	**0.99998±0.00001**	**100%**	**0.001%±0.001%**
100	0.9977 ± 0.0007	68.10% ± 6.19%	0.07% ± 0.01%	**0.99999±0.00002**	**100%**	**0.002%±0.002%**
150	0.9986 ± 0.0005	74.95% ± 4.68%	0.06% ± 0.00%	**0.99998±0.00002**	**100%**	**0.001%±0.001%**
500	0.9997 ± 0.0001	93.10% ± 3.63%	0.07% ± 0.00%	**0.99999±0.00001**	**100%**	**0.01%±0.01%**
1000	0.9999 ± 0.0001	97.10% ± 1.92%	0.06% ± 0.01%	**0.99998±0.00002**	**100%**	**0.02%±0.01%**
2000	0.9999 ± 0.0001	96.95% ± 1.73%	0.07% ± 0.01%	**0.99999±0.00001**	**100%**	0.07%±0.01%
All	0.9995 ± 0.0002	87.75% ± 2.51%	0.06% ± 0.02%	**0.99998±0.00002**	**100%**	0.08%±0.00%

**Table 6:** Detection result of random push attacks(with 100 bots) when the target items varies.

	SVD-based method			RD-TIA		
Target items	AUC	Detection Rate	False Alarm Rate	AUC	Detection Rate	False positive rate
1	0.9999 ± 0.0001	96.95% ± 1.73%	0.07% ± 0.01%	**0.99999±0.00001**	**100%**	**0.003%±0.002%**
2	0.9999 ± 0.0001	97.45% ± 1.32%	0.06% ± 0.01%	**0.99997±0.00001**	**100%**	**0.005%±0.003%**
5	0.9999 ± 0.0000	97.75% ± 1.37%	0.07% ± 0.01%	**0.99992±0.00002**	**100%**	**0.016%±0.005%**
10	0.9997 ± 0.0002	92.20% ± 4.50%	0.07% ± 0.01%	**0.99989±0.00002**	**100%**	**0.023%±0.004%**
20	0.9987 ± 0.0002	65.15% ± 4.61%	0.08% ± 0.02%	**0.99983±0.00000**	**100%**	**0.033%±0.000%**

Table 7 shows the detection result of average push attacks with an attack size of 100 when the number of rated items in each profile varies. All three parameters of *RD-TIA(a)* are better than *SVD*-based method. From the results it can be seen that our technique outperforms *SVD*-based algorithms.

**Table 7:** Detection result of average push attacks (with 100 bots) when the number of filler items vary.

	SVD-based method			RD-TIA		
Rated items	AUC	Detection rate	False Positive Rate	AUC	Detection Rate	False Positive Rate
20	0.9614 ± 0.0207	3.65% ± 2.72%	0.18% ± 0.01%	**0.99513±0.00131**	**100%**	**0.005%±0.003%**
50	0.9735 ± 0.0078	14.10% ± 5.54%	0.17% ± 0.01%	**0.99999±0.00001**	**100%**	**0.001%±0.001%**
100	0.9582 ± 0.0088	6.45% ± 2.58%	0.18% ± 0.01%	**0.99998±0.00001**	**100%**	**0.003%±0.003%**
150	0.9324 ± 0.0121	1.85% ± 1.46%	0.20% ± 0.01%	**0.99999±0.00001**	**100%**	**0.002%±0.002%**
500	0.6844 ± 0.0101	0	0.24% ± 0.01%	**0.99999±0.00002**	**100%**	**0.002%±0.003%**
All	0.5141 ± 0.0054	0	0.22% ± 0.02%	**0.99999±0.00002**	**100%**	**0.002%±0.003%**

*RD-TIA(a)* is a group detection method. Since attack profiles work together to perform an attack, *RD-TIA(a)* can capture group features of profiles. All profiles rate the target item with maximum or minimum score to push or nuke a target item. The idea is first find the target item of an attack, and then filter out genuine profiles rate on the target item. Metric *RMDA* and *DegSim* are used to concentrate all suspicious profiles into a set. In the rating matrix consist of suspicious profiles, *TIA* method is used to find target items, if the target items are found right, all profiles that rate on the target item with maximum or minimum score can be found, this is why the detection rate is almost 100%. The false positive rate is low because the matrix is sparse. False negatives exist because attack profiles are lost in first phase of *RD-TIA(a)*, so threshold value of *RMDA* and *DegSim* are set as low as possible.

#### 4.2.4 Discussions

From the results we notice that the detection rate of random attack is higher than that of average attack. The false positive rate of nuke attack is lower than that of push attack. Table 8 shows the rating distribution in ML100K Dataset. Rating score of 1 is 6.07% of the rating distribution, whereas rating score of 5 is 21.07% of the rating distribution. Due to this the false positive rate is lower in nuke attacks as compared to push attacks. When nuke attacks are carried out a target item is usually rated low (usually 1), however due to the lower distribution of rating 1’s within the dataset, it is easier to differentiate a nuke attack as compared to a push attack whereby a target item is rated high (usually 5) and the distributions of rating 5 are higher. On the other side, *AUC* values of nuke attacks are lower than that of push attacks. The false positive rate of single-targeted detection is lower than that of multi-targeted item detection; while the *AUC* values of single-targeted detection are higher than that of multi-targeted item detection. The detection rate reaches 100% in most situations with some false positives, except that there exist some false negatives in average detection. There exist false negatives average detection when filler size is 3%, both in single-targeted average detection and multi-targeted.

**Table 8:** Proportion of five ratings in ML100K Dataset.

Value	Count	Percentage
1	4853	6.07%
2	9185	11.48%
3	21811	27.26%
4	27294	34.12%
5	16857	21.07%

Table 9 shows the detection rate of single average detection when filler size is 3% when the attack size varies. Table 10 shows the detection rate of multi-targeted detections when filler size is 3% and the target items vary.

**Table 9:** The detection rate of single average detections.

Attack size	Single average push	Single average nuke
19	99.995%	99.989%
38	99.995%	99.989%
57	99.995%	99.995%
75	99.992%	99.995%
94	99.989%	99.989%
113	99.985%	99.990%
132	99.989%	99.993%

**Table 10:** The detection rate of multi average detections.

Target items	Multi average push	Multi average nuke
1	99.990%	99.995%
2	99.990%	99.993%
3	99.993%	99.997%
4	99.993%	99.996%
5	99.994%	99.994%
6	99.995%	99.997%
7	99.992%	99.995%
8	99.994%	99.991%
9	99.994%	99.993%
10	99.993%	99.992%

### 4.3 Performance of *RD-TIA(b)*

In this section, we test the detection of group attacks, including segment and bandwagon attack models. In order to achieve better attack effectiveness, attackers combine different attack models to make attack profiles more similar to genuine profiles, as described in [25]. We call this the “combined attack models”. We test our method using single attack model and combined attack models. In single attack model, there is only one attack model in an attack; while in a combined attack, there are two different types attack models combined together. The results of detection on single model attacks and combined model attacks are shown in the following sections.

#### 4.3.1 Single Attack Model Detection

In this section, we will show experimental results using *RD-TIA(b)* detection method on single model attacks, where all of the attacks are push attacks. Attack profiles are somewhat similar to the average attack model except for the selected set. Attack profiles are generated as follow: in the selected set, 1% of the most rated items in the datasets are chosen, these items are rated as maximum rating; the ratings for filler items are distributed around the average rating for each item; the target item is randomly chosen and rated with the maximum rating.

**Fig 8:**
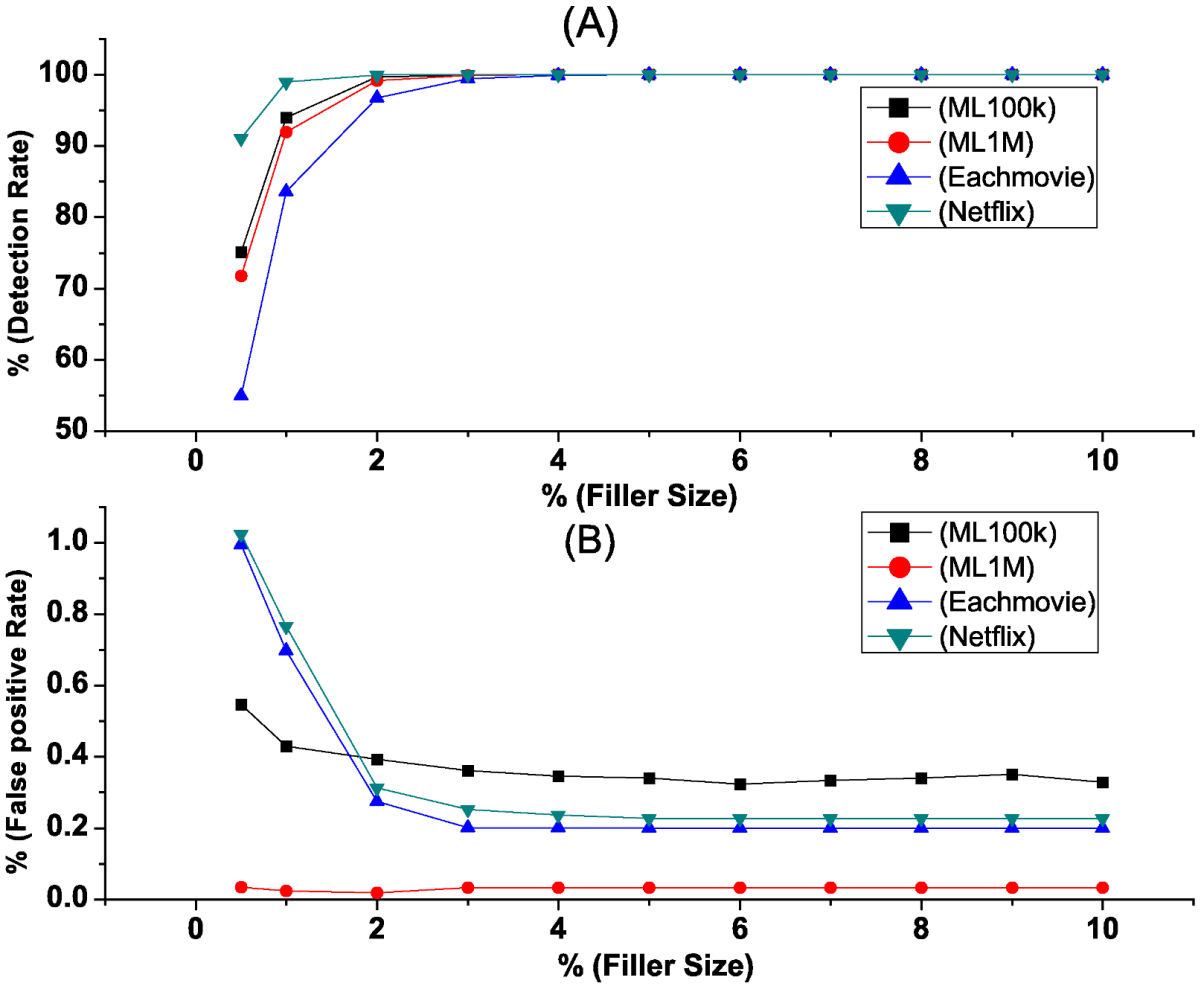
Detection rate and false positive rate when the attack size is 5% and filler size varies in different datasets. Detection rate and false positive rate are detected when the attack size is 5% and filler size varies using four different datasets, including MovieLens 100k Dataset, MovieLens 1M Dataset, Eachmovie Dataset and Netflix sub-Dataset.

Fig 8 shows the detection rates and false positive rates of the proposed method while facing group attacks on four different datasets. Fig 8(A) is the detection rates when the attack size is 5% and filler size varies. Fig 8(B) shows the false positive rates of the detection. We find that the detection rates increase along with the increase in filler size. The detection rates reach close to 100% when filler size reaches 4%. The false positive rates, on the other hand, consistently stay below 0.5% when the filler size is greater than 2%, regardless of attack sizes.

**Fig 9:**
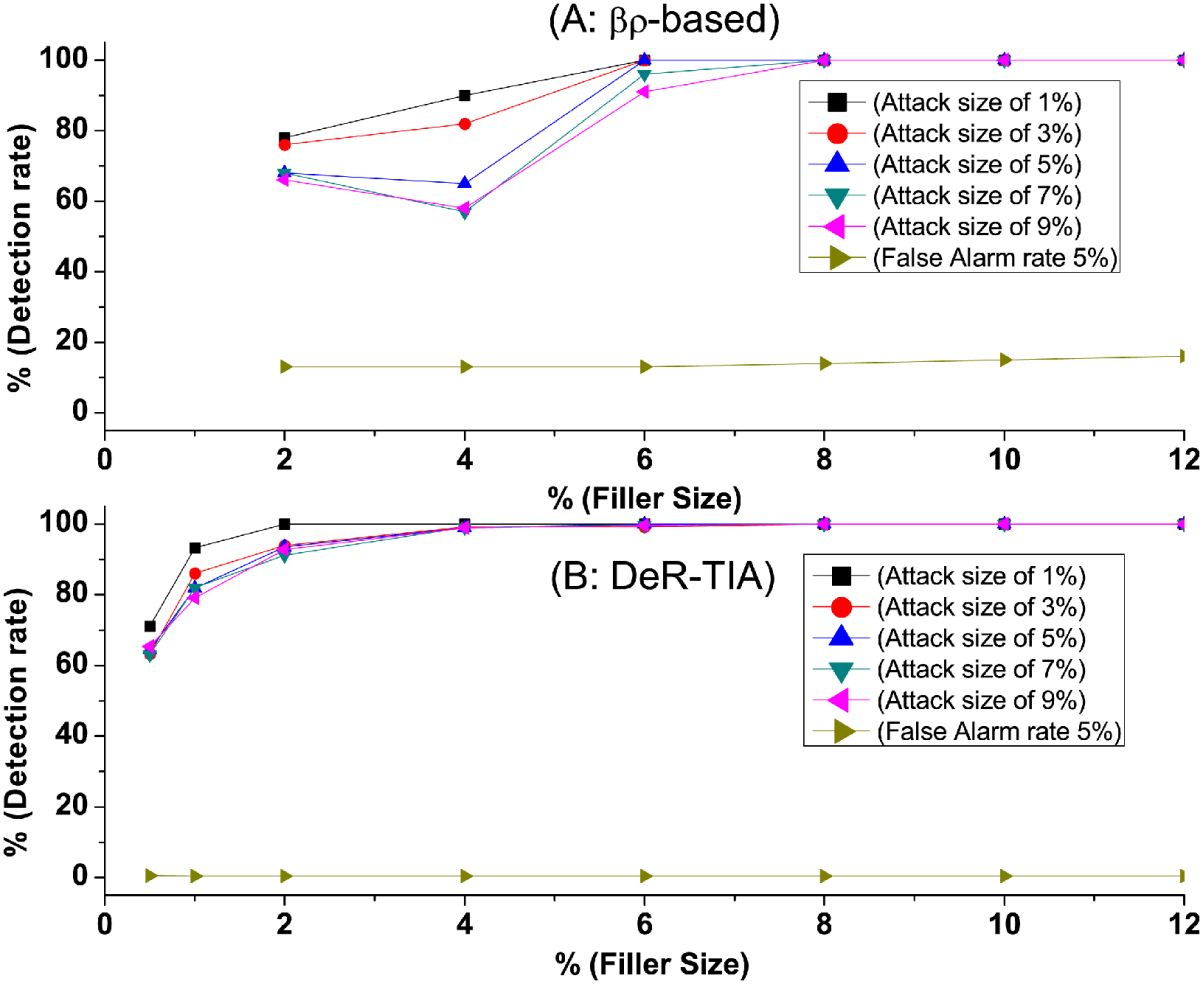
Detection rate of single-targeted attacks when attack size and filler size varies. There is only one target item in each attack. Comparison of detection rate when attack size and filler size varies.

In the second test, we compare our method with the state-of-the-art unsupervised *βρ*-based method in [31] using ML100k Dataset when the selected set is none, that is, there is only one target item in an attack profile, and the filler size is 5% and the attack size varies from 1%, 3%, 5%, 7% to 9%. Fig 9 shows the detection rates of *βρ*-based method (A) and our method (B) when facing single-targeted push attacks. In our method (B), the detection rate reaches almost 100% when the filler size is greater than 4%. The false positive rates are lower.

**Fig 10:**
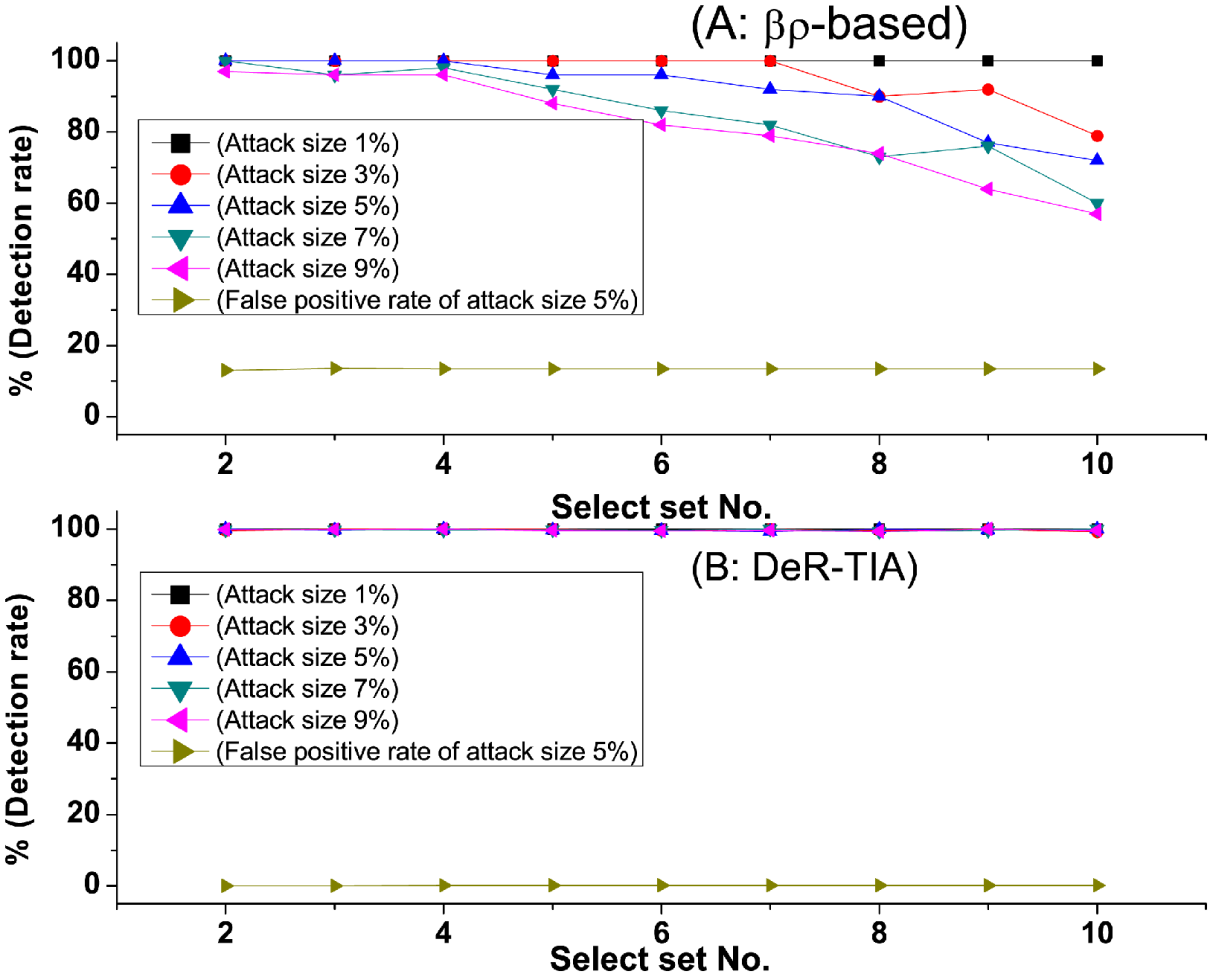
Detection rate of group attacks when the attack size and target items vary. There are multi-target items in each attack. Comparison of detection rate when attack size and filler size varies.

In the third test, we compare *βρ*-based method and our method using ML100k Dataset when the selected set varies from 2 to 10. Fig 10 shows the detection rates of *βρ*-based method (A) and our method (B) when facing group push attacks. In our method, the detection rate reaches almost 100%. The false positive rates are below 0.5%, which is low. Our method reaches higher detection rates and lower false positive rates.

#### 4.3.2 Combined Attack Models Detection

In this section, we will show experiment results using *RD-TIA(b)* detection method on combined model attacks. Three single attack models, random, average and bandwagon attack models, and two combined attack models, random&bandwagon and average&bandwagon attack models are tested in this section. All the attacks in this section are push attacks, with attack size is 10% and the filler size varies from 1% to 50%. ML100K Dataset is used in the flowing test.

**Fig 11:**
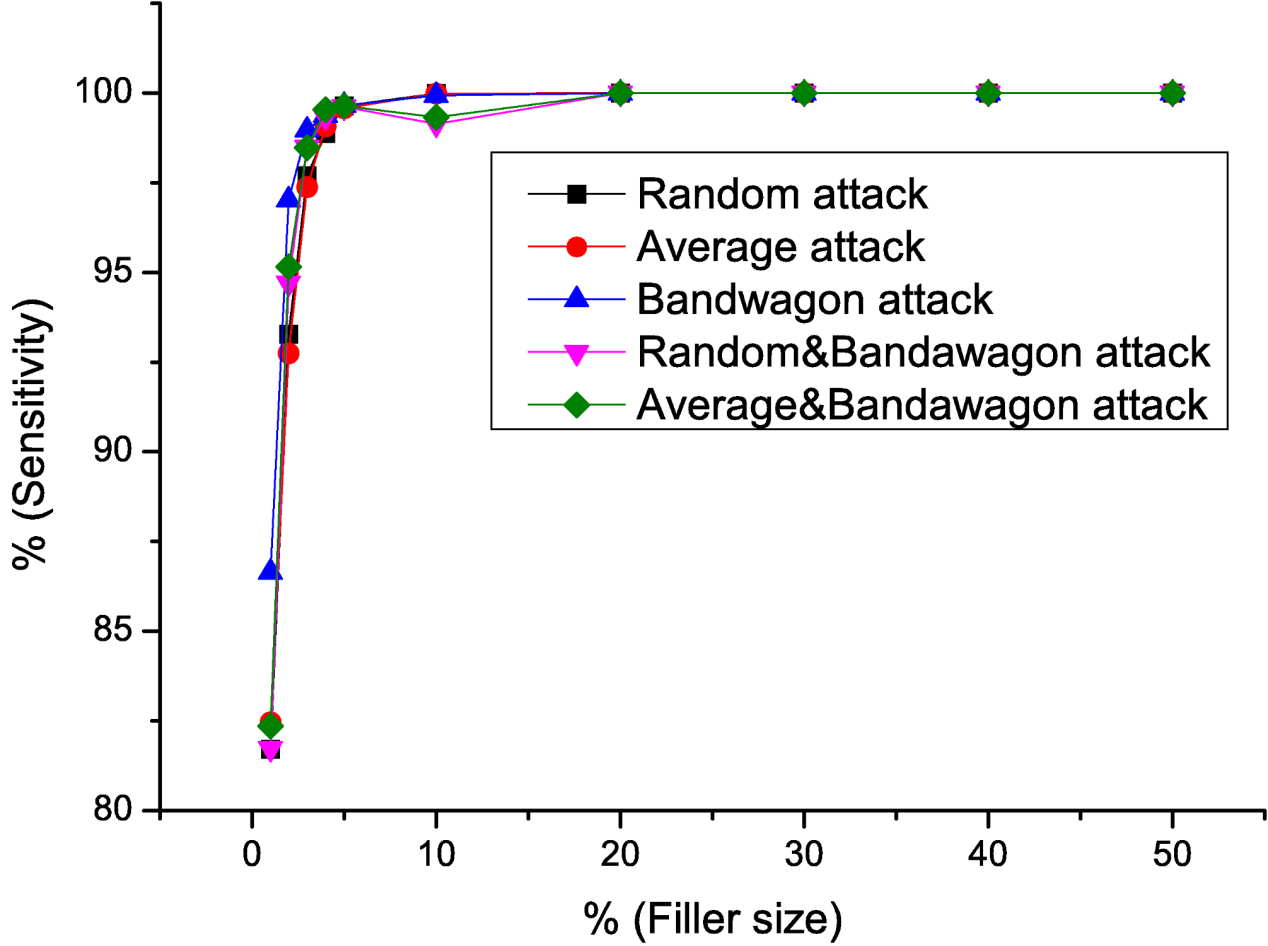
Sensitivity of different attack models when filler size varies using *RD-TIA(b)*. Detecting result of different attack models when filler size varies using *RD-TIA(b).*

**Fig 12:**
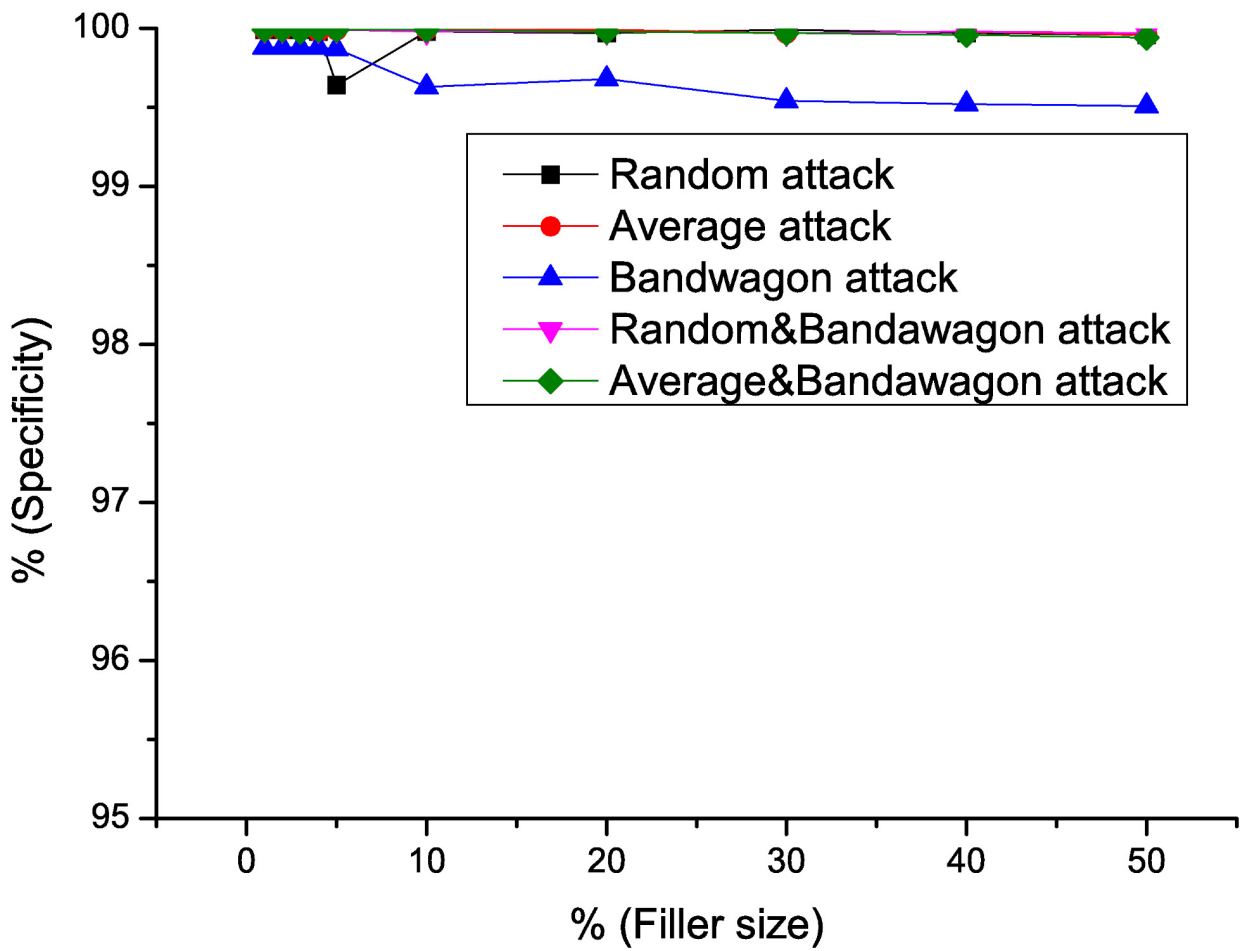
Specificity of different attack models when filler size varies using *RD-TIA(b)*. Detecting result of different attack models when filler size varies using *RD-TIA(b).*

**Fig 13:**
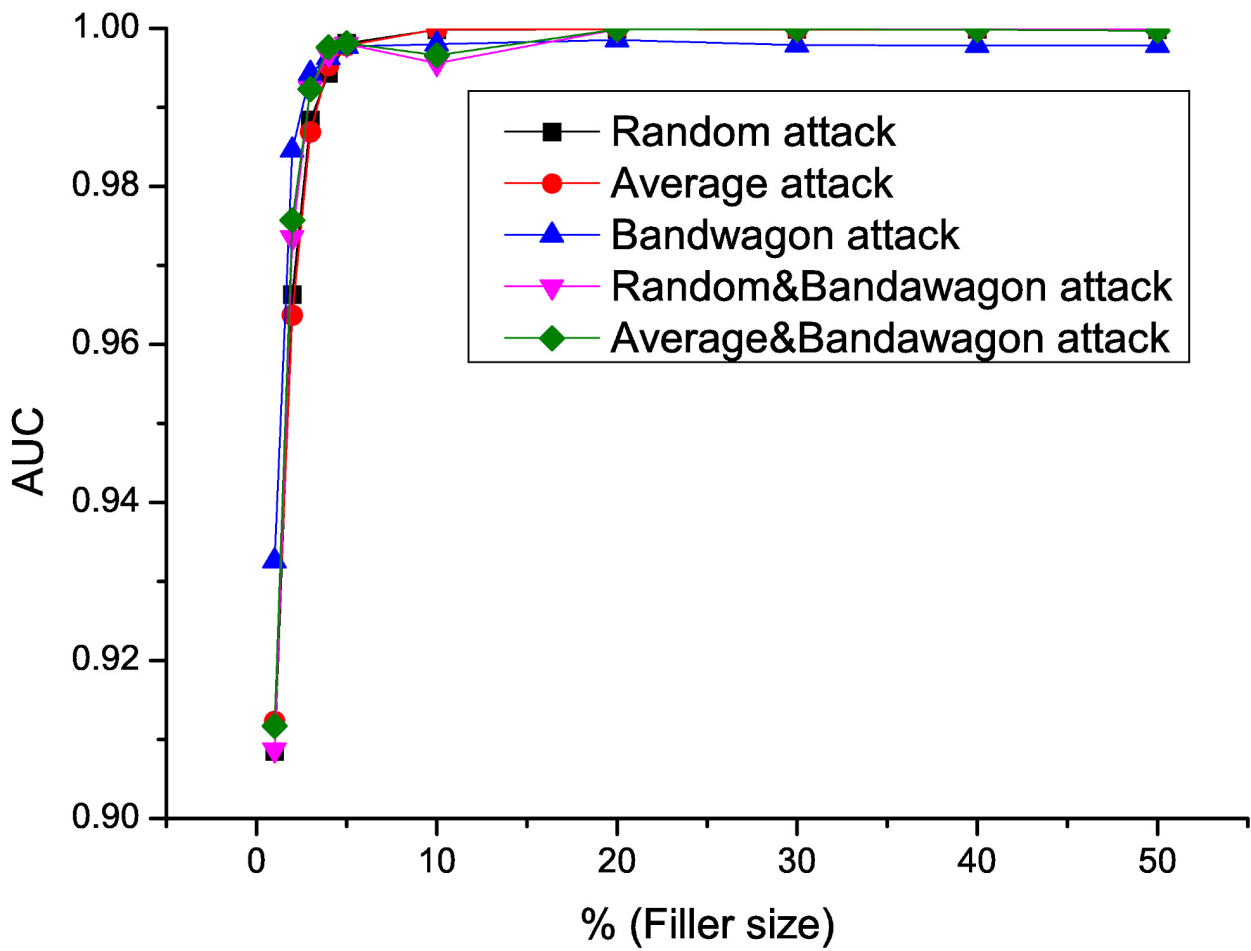
AUC value of different attack models when filler size varies using *RD-TIA(b)*. Detecting result of different attack models when filler size varies using *RD-TIA(b).*

Sensitivity, Specificity, *AUC* value of the detecting results using *RD-TIA(b)* against five attack models are shown in Figs 11–13. We can see from the results that the specificity of all the tests is high. According to Eq (7), we know that there are only a small number of false positives in the result. There are a common characteristic in five detection results, when the filler size of attacks is greater than 3%, the sensitivity of the results gets better, which means only a small number of false negatives exist in the results by Eq (6). In Table 3, we can see that all of the datasets we use are very sparse. Metric *DegSim*′ does not reflect the rating distribution well when the filler size is smaller than 3%. Detecting results using *RD-TIA(b)* are not as good when the filler size is smaller than 3%. The experiments in this section show that our proposed method *RD-TIA(b)* can efficiently detect combined model attacks. The performance of detecting combined profiles is better when the filler size is greater than 3%.

## 5 Conclusions

In this paper, we present *RD-TIA*, a novel unsupervised shilling attack detection structure that use profile rating patterns and group feature of attack profiles. Two different statistical metrics are used to detect attackers based on their rating patterns. We proposed two detection algorithms based on *RD-TIA* structure. In *RD-TIA(a)*, the threshold value is set to find suspicious profiles. Tests show that using the threshold method can achieve a high-accuracy result, using extra knowledge for the threshold value, but there are restrictions in detecting more complex attack models. So we propose another detection method based on *RD-TIA* structure. In *RD-TIA(b)*, a new metric *DegSim*′ is proposed based on *DegSim*. Tests show that *RD-TIA(b)* is useful to detect segment attacks and combined attack models. *TIA* methods are used to filter out genuine profiles based on the suspicious profiles we get in both of the methods.

Table 11 shows detection result when there are no attacks injected using ML100K by *RD-TIA(a)* and *RD-TIA(b)*. Table 12 shows the comparison of two *RD-TIA* methods. *RD-TIA(a)* needs more knowledge before detection, has high accuracy and consumes less time, but detects only random and average attacks; *RD-TIA(a)* needs less knowledge before detection, detect random, average, bandwagon and segment attacks, but the accuracy is not as high as *RD-TIA(b)* and consumes more time.

**Table 11:** Detection result when there are no attacks injected using ML100K.

Methods	Profiles that misjudged as attackers
RD-TIA(a)	195, 219, 358
RD-TIA(b)	46, 112, 126, 166, 206, 260, 507, 519, 531, 578, 609, 626, 724, 782, 841

**Table 12:** Comparison of two *RD-TIA* attack detection methods.

	RD-TIA(a)	RD-TIA(b)
**Detect models**	random and average attacks	random, average, bandwagon and segment attacks
**Knowledge need**	more	less
**Accuracy**	higher	lower
**Time consuming**	less	more

The algorithms we proposed in the paper detects attackers and does not require attacking profiles as training data, which means it is immune to missing values, but there are some limitations on the *RD-TIA* methods. The first limitation is that we need to know the features of metrics we use in the structure, for example, we should know he *RDMA* value is higher and the *DegSim* value is lower in attack profiles. This requires some knowledge of the rating distribution information of the dataset. The other limitation is with the time consumed as the scale of the dataset increases. We obtained the *DegSim* value by calculating the similarity with other profiles. The computational cost of the metric *DegSim* is square stage growth with the number of profiles. Based on the above limitations, in the future, we will try to introduce supervised learning methods so that we do not have to know the features of the metrics. We will try different metrics using the structure and find a way to reduce time complexity. In the future, we will extend our techniques to detect more complex attack models.
